# Two faces of bivalent domain regulate VEGFA responsiveness and angiogenesis

**DOI:** 10.1038/s41419-020-2228-3

**Published:** 2020-01-30

**Authors:** Jiahuan Chen, Xiaodong Liang, Shasha Zhang, Shiyan Wang, Sara P. Garcia, Pengyi Yan, Huijing Yu, Zixuan Li, Li Liu, Fang Zhang, Weiting Wei, Huangying Le, Yan Zhang, Guo-cheng Yuan, Sun Chen, Yingwei Chen, Kun Sun, William T. Pu, Bing Zhang

**Affiliations:** 1 Department of Pediatric Cardiology, Xin Hua Hospital, School of Medicine, Key Laboratory of Systems Biomedicine, Shanghai Center for Systems Biomedicine, Shanghai Jiao Tong University, Shanghai, 200240 China; 2 School of Life Science and Food Engineering, Huaiyin Institute of Technology, Huaian, 223003 China; 3 Department of Biostatistics and Computational Biology, Dana-Farber Cancer Institute and Harvard T.H. Chan School of Public Health, Boston, MA 02215 USA; 4 Renji-Med Clinical Stem Cell Research Center, Renji Hospital, School of Biomedical Engineering, Shanghai Jiao Tong University, Shanghai, 200127 China; 5 Department of Cardiology, Boston Children’s Hospital, Boston, MA 02115 USA; 6 Harvard Stem Cell Institute, Cambridge, MA 02138 USA

**Keywords:** Biochemistry, Molecular biology

## Abstract

The bivalent domain (BD) at promoter region is an unique epigenetic feature poised for activation or repression during cell differentiation in embryonic stem cell. However, the function of BDs in already differentiated cells remains exclusive. By profiling the epigenetic landscape of endothelial cells during VEGFA (vascular endothelial growth factor A) stimulation, we discovered that BDs are widespread in endothelial cells and preferentially marked genes responsive to VEGFA. The BDs responsive to VEGFA have more permissive chromatin environment comparing to other BDs. The initial activation of bivalent genes depends on RNAPII pausing release induced by EZH1 rather than removal of H3K27me3. The later suppression of bivalent gene expression depended on KDM5A recruitment by its interaction with PRC2. Importantly, EZH1 promoted both in vitro and in vivo angiogenesis by upregulating EGR3, whereas KDM5A dampened angiogenesis. Collectively, this study demonstrates a novel dual function of BDs in endothelial cells to control VEGF responsiveness and angiogenesis.

## Introduction

In the past 30 years, histone modifications and chromatin-modifying enzymes emerged as essential regulators of gene expression^1^. Most functional genomic regions featured with covalent histone modifications link to gene activation (H3K4me3 and H3K27ac) or repression (H3K27me3 and H3K9me3)^1,2^. In 2006, Bernstein et al.^3^ analyzed the chromatin occupancy of a series of histone modifications in mouse embryonic stem (ES) cells and found that 75% of H3K27me3 promoter regions were also marked by H3K4me3. This class of promoter regions with both activating H3K4me3 and repressive H3K27me3 marks was named the bivalent domain (BD). Subsequent studies showed that BDs were found widely in tissues of higher vertebrates, such as zebrafish and mammals, but not in lower vertebrates and arthropods, such as *Xenopus* and *Drosophila*^4^.

In ES cells, bivalent genes are enriched for developmental transcription factors and expressed at low levels. Upon lineage-specific differentiation, some bivalent genes lost H3K27me3 and became fully activated, whereas others lost H3K4me3 and were silenced^3^. These observations suggested that BDs in ES cell provide a permissive environment for developmental genes to turn on or off in response to developmental cues. In mature, differentiated tissues, BDs are also present, but their function is not well understood. Are they merely leftover from cell commitment, or do they possess other important functions?

The generation of BDs requires both Trithorax and Polycomb complexes. H3K4me3 of BDs is established by two types of Trithorax methyltransferases, MLL and SET1A/B, which are recruited to CpG islands by CXXC domain-containing proteins^5^. H3K4me3 demethylases of the Jarid/KDM5 classes, including KDM5A (Jarid1a) and KDM5B (Jarid1b), compete with the methyltransferases and erase H3K4me3 (ref. ^6^). On the other hand, the repressive mark of H3K27me3 is created and maintained by Polycomb complexes, including Polycomb complex 1 (PRC1) and Polycomb complex 2 (PRC2). The methyltransferase subunit of PRC2 is either EZH1 or EZH2 (ref. ^7^). Removal of H3K27me3 at BDs requires the demethylase UTX, a component of MLL3/4 complexes. Intriguingly, PRC2 is able to recruit KDM5A to BD and mediate the demethylation of H3K4me3, illustrating a shared machinery between active and repressive mark. Loss or gain of either H3K27me3 or H3K4me3 is a major mechanism reported previously for activating or silencing the transcription of bivalent genes^8,9^. However, it was still unclear if the BD and its catalytic machineries involve in the regulation of transcription response to environmental stress, such as VEGFA (vascular endothelial growth factor A) stimulation.

VEGFA governs vasculogenesis and angiogenesis. Anti-VEGFA therapy emerged as an important approach to treat certain cancers and age-related macular degeneration^10^. VEGFA interacts with VEGFA receptors, mainly VEGFA receptor 2 in endothelial cells, to promote endothelial cell proliferation, migration, and permeability. We and others showed that VEGFA induces rapid and pervasive gene expression changes in endothelial cells. For instance, our recent study showed that VEGFA promoted gene expression and angiogenesis by stimulating acetylation of ETS1 or increasing genome-wide deposition of H3K27ac. Despite these advances, many gaps remain in the understanding of how VEGFA modulates the epigenome and regulates gene transcription.

## Results

### BD associated with VEGFA-responsive genes

**Fig. 1:**
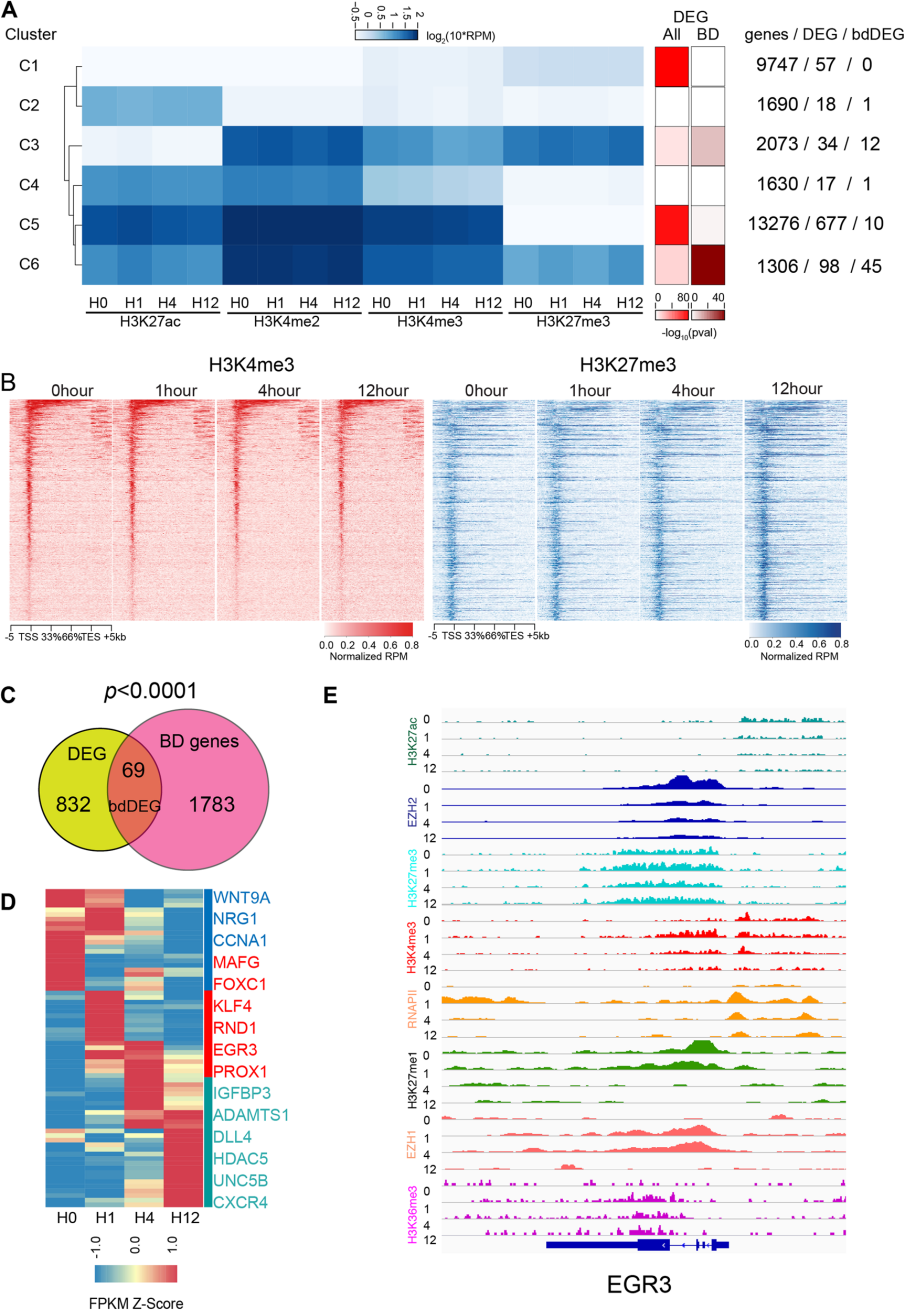
DEGs were enriched at BDs. **a** The correlation between histone clusters and DEG. The left heatmap is the *K*-means clusters of four different histone marks (H3K4me3, H3K4me2, H3K27ac, and H3K27me3). The middle and the right heatmaps represent the enrichment of DEGs in the set of all genes and BD genes, respectively, at divergent histone clusters by Fisher’s exact test. The text on the right side described the number of TSS, DEG, and bdDEG within each cluster. **b** Tag heatmaps of H3K4me3 and H3K27me3 at all BDs called by H3K27me3 and H3K4me3 peak overlap. **c** Venn diagram showing the overlap between DEGs and BD genes. We found 69 DEGs were marked by BD, a highly significant enrichment compared to expectations of randomness. Chi-squared test: *P* < 0.05 indicated significance. **d** Heatmap of bdDEGs from RNA-seq, grouped into three clusters: downregulated genes (blue), early-upregulated genes (red), and late-upregulated genes (jasper). Representative genes within each cluster are listed on the right. **e** Snapshot of Integrative Genomics Viewer of histone marks, RNAPII, and transcribed RNA near *EGR3*, a bdDEG that was rapidly and transiently upregulated by VEGFA.

Our model system consisted of human umbilical vein endothelial cells (HUVECs) that were cultured overnight in low serum and no VEGFA, and then stimulated for 12 h with 50 ng/ml VEGFA. Samples were collected at 0 (unstimulated), 1, 4, and 12 h. To interrogate if the integrated pattern of histone modifications configured chromatin responsiveness to VEGFA, we used chromatin immunoprecipitation followed by high-throughput sequencing (ChIP-seq) to profile the genomic occupancy of multiple histone modifications, including H3K4me2 (a mark of both active promoters and enhancers), H3K4me3 (a mark of active promoters), H3K27ac (a mark of active enhancers and promoters), and H3K27me3 (a mark of repressed enhancers and promoters). Using our previously reported RNA sequencing (RNA-seq) expression profiles for this experimental system^11^, we identified 29,722 expressed genes (RPKM ≥ 1 at any of the four tested time points). At the promoters of these genes (TSS ± 1 kb), the ChIP-seq signals of these four histone modifications were grouped into six clusters using *K*-means clustering and the optimal *K* was determined by Silhouette algorithm (Methods section). Each cluster had >1000 genes (Fig. 1a, Supplementary Table 1). Cluster C1 did not have significant signal at the promoter for any of the profiled histone modifications. C2 had weak H3K27ac but no other active histone marks. C3–6 had at least two active histone marks present. Among them, C5, containing high signals for all three activating histone marks, was the most dominant cluster (13,276 TSSes). Importantly, there were a total of 3379 promoters in C3 and C6 that were occupied by both H3K4me3 and H3K27me3, suggesting they were BDs (Fig. 1a, Supplementary Table 1).

In the same model system, our previous studies identified 901 genes that were significantly changed by VEGFA (differentially expressed genes: DEG, Supplementary Table 2). To further probe the association of these epigenetic patterns with DEG at transcriptional start site (TSS), we calculated the number and enrichment score of DEG in each histone modification cluster (Fisher’s exact test; see Methods section; Fig. 1a, right side). DEGs were most highly enriched in C5, the cluster with strong signals for all of the active histone marks. Surprisingly, C3 and C6, which had both active and repressive marks, were also enriched with DEGs (*P* = 1.0009 × 10^−11^ and 1.935 × 10^−16^, respectively, Fisher’s exact test). This suggested a possibility that the bivalent epigenetic signature contributed to the responsiveness of VEGFA. To further test this hypothesis, we used a more pervasive approach based on the co-occurrence of H3K27me3 and H3K4me3 at the same promoter to identify BD promoters. This identified 1851 BD promoters (Supplementary Table 1). Histone tag heatmaps at the promoters showed co-occurrences of both H3K4me3 and H3K27me3 (Fig. 1b), and 81.79% of these promoters overlapped with C3 and C6 (Supplementary Fig. 1a). The expression level of bivalent genes was lower than all expressed genes (BD vs all expressed genes, *P* < 2.2 × 10^−16^, Wilcoxon rank-sum test, Supplementary Fig. 1b), consistent with observations in other cell lineages^3^. When considering TSSs targeted by H3K4me3 with or without H3K27me3, genes with only H3K4me3 showed significantly higher fragments per kilobase of exon model per million reads mapped (FPKM) than bivalent DEGs (bdDEG; *P* = 0.007671) and BDs (*P* < 2.2 × 10^−16^; Supplementary Fig. 1c). Therefore, we used these 1851 peak-overlapping BD promoters for the following analysis.

We observed that there were more DEGs with promoter BDs than expected by random chance (69/901; *P* < 0.001, Chi-square test; Fig. 1c, Supplementary Fig. 1c). Some bdDEGs had key functions in vascular identity and homeostasis (representative genes are listed on the right side of Fig. 1d). For instance, the Notch pathway ligand DLL4 antagonizes VEGF’s proangiogenic effect and promotes tip cells to stalk cells transition^12^. *FOXC1* is an upstream regulator of *DLL4*^13^. Both *DLL4* and *FOXC1* have bivalent promoters, suggesting that bivalency may play an essential role in NOTCH signaling (Fig. 1d). A subset of endothelial cell transcription factors also had bivalent promoters, including *KLF4*, a master regulator of endothelial cell identity and hemodynamics^14^ (Fig. 1d) and *EGR3*, which has multiple functions in endothelial cell growth, sprouting, and tube formation and was upregulated >100-fold after 1 h of VEGFA stimulation (Fig. 1d, e). Together, enrichment of BDs at the promoters of DEGs and the key functions of bdDEGs in endothelial biology indicated that BD may have an irreplaceable role in mediating the proangiogenic effect of the VEGFA pathway.

### Epigenetic signature of bdDEG

**Fig. 2:**
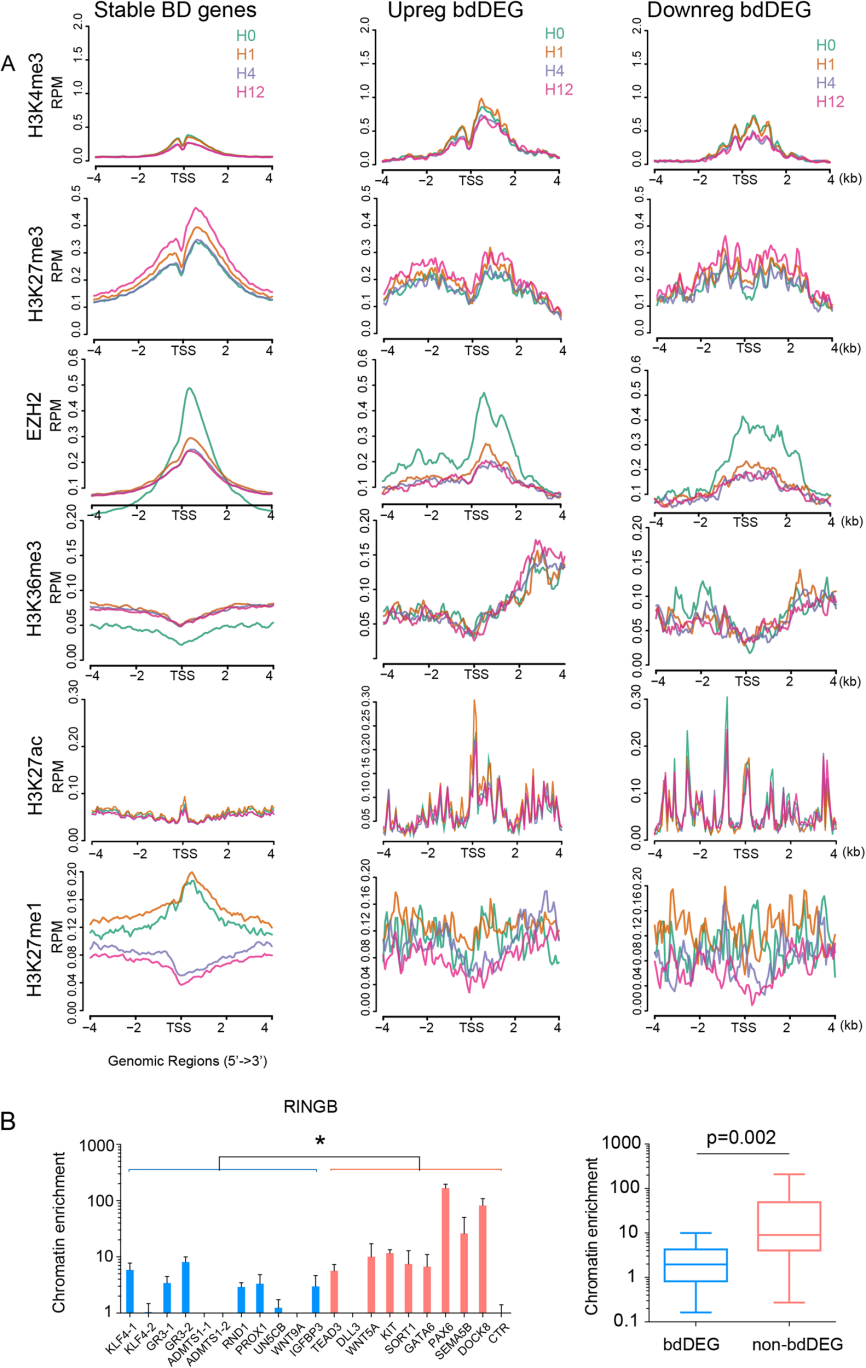
Chromatin features of bdDEG at TSS. **a** Aggregation plots of histone modifications near promoters of all BD genes (left) and upregulated (middle) or downregulated (right) bdDEGs. **b** RINGB chromatin occupancy at BD genes that were (bdDEG, blue) or were not (non-bdDEG, megenta) differentially expressed, as measured by ChIP-qPCR. Box plot on the right summarizes the chromatin enrichment of RINGB at each class of genes; *n* = 4 for each gene loci, Mann–Whitney *U* test.

To further understand the mechanism underlying alternative expression of bdDEG, we compared the epigenetic features of up- and downregulated bdDEGs to stable BD genes that were not changed by VEGF (Fig. 2a). bdDEG (middle and right columns) showed stronger H3K4me3 and weaker H3K27me3 at their TSSs comparing to stable BD genes (left column). Further separating the upregulated bdDEG to early (gene upregulated at 1 h) and late upregulation (gene upregulated at 4–12 h) group demonstrated a significant and early induction of H3K4me3 and H3K27me3 in early but not in late-upregulated bdDEG group (Supplementary Fig. 2). Upregulated bdDEG also had stronger H3K36me3, a mark of active mRNA transcription, at their gene bodies (Fig. 2a). Surprisingly, H3K27ac, a canonical active histone modification on the same lysine residual of histone H3 with H3K27me3, was present at bdDEGs with remarkably higher signals than at non-bdDEGs. H3K27ac had very little overlap with H3K27me3, which was consistent with the fact that they are mutually exclusive in biochemistry (Fig. 2a). Both up- and downregulated bdDEG had elevated H3K27ac, but the pattern was different, with upregulated bdDEGs having a sharper H3K27ac peak near the TSS and overlapping with H3K4me3, and downregulated bdDEG having a more diffusive H3K27ac peak across up- and downstream of TSS (Fig. 2a). H3K27ac upregulation at 1 h after VEGFA treatment was mainly contributed by early upregulated bdDEGs (Supplementary Fig. 2). In line with the presence of multiple active histone marks at BD, H3K27me1 with a recently suggested role in positively regulating transcription, also weakly presented at all BD genes (Fig. 2a)^15,16^. Early compared to late-upregulated bdDEGs demonstrated more dramatic augment in H3K27me1 deposition (Supplementary Fig. 2).

Of note, promoter occupancy by EZH2, the methyltransferase catalytic subunit of PRC2, markedly decreased after VEGFA treatment in BD genes (Fig. 2a). PRC1, the first discovered Polycomb complex, at many loci, was found to cooperate with PRC2 in setting up the BD^4^. To determine if PRC1 has a similar function to maintain the bivalency in the endothelial cells as well, we measured the chromatin occupancy of RINGB, a catalytic subunit of PRC1 that ubiquitinates H2AK119 to deepen the transcription repression^17,18^. Less RINGB occupancy at bdDEG (genes with blue bars) was observed compared to that at unresponsive BD (genes with carmine bars, Fig. 2b). Together, these results suggest that bdDEGs had a more permissive chromatin state for transcription manifested by higher occupancy of active histone marks (H3K27ac, H3K36me3, andH3K4me3) and less occupancy of repressive marks (H3K27me3 and PRC1) compared to other BD genes that were not differentially expressed.

### EZH1 mediated the activation of bivalent genes

**Fig. 3:**
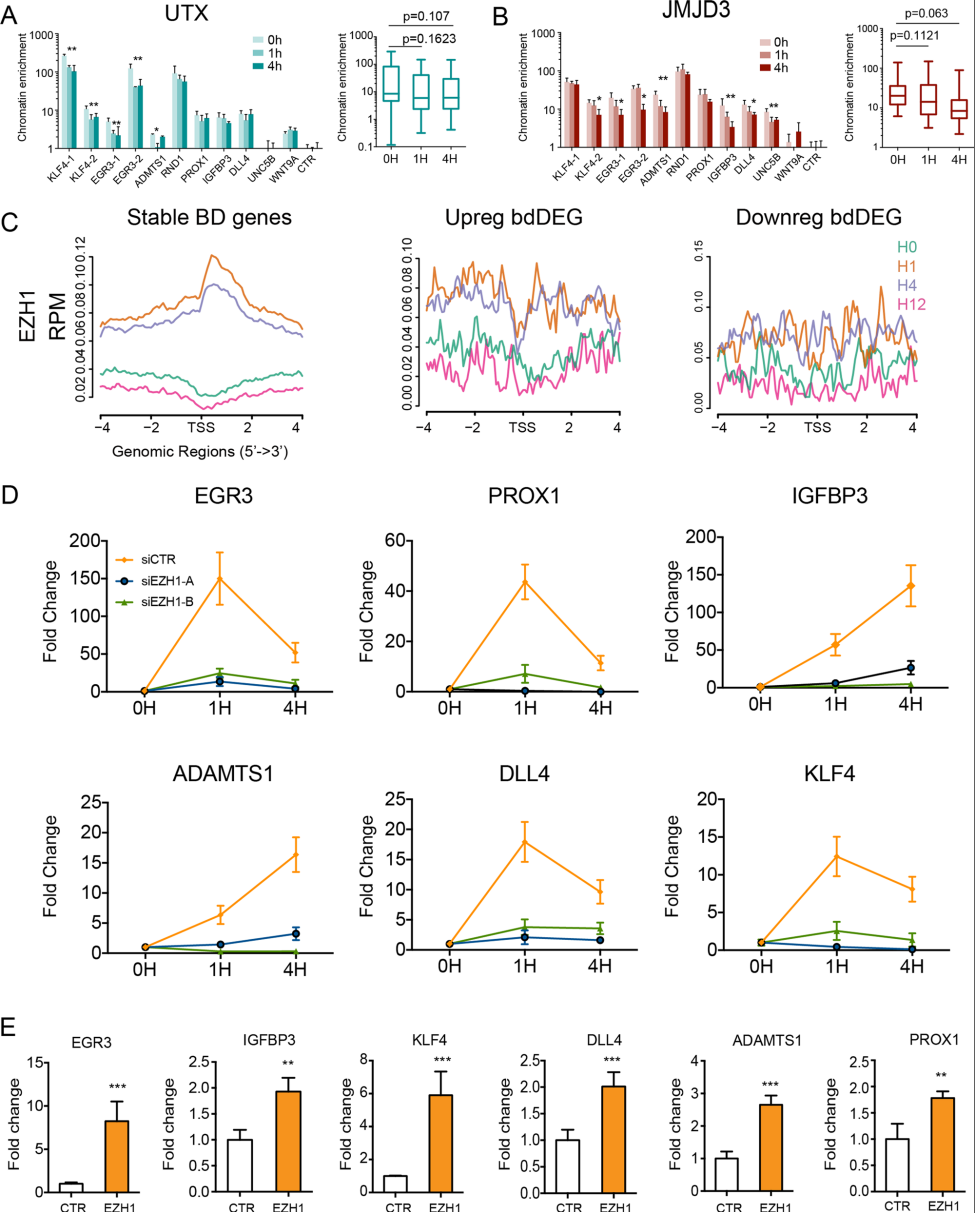
VEGFA treatment increased the occupancy of EZH1 complex at upregulated bdDEG. **a**, **b** UTX **a** and JMJD3 **b** chromatin occupancy at bdDEG, as measured by ChIP-qPCR. The box plot summarizes the chromatin enrichment of UTX and JMJD3 at each time point; *n* = 4, *P < 0.05, **P < 0.01. Mann–Whitney *U* test in summary panels. **c** Aggregation plot of EZH1 near proximal promoters of all BD genes separated into stable, upregulated, and downregulated groups. **d** Inhibitory effect of *EZH1* siRNA on bdDEGs activation as measured by RT-qPCR. EZH1 in HUVEC was suppressed by *EZH1* siRNA and then treated with VEGFA for 1–4 h. *EZH1* knockdown abolished the activation of six genes activated by VEGF. Plots show mean ± SD; *n* = 4, two-tailed Student’s *t*-test: *P < 0.05 compared to control at the same time point. **e** EZH1 upregulated bdDEG expression in HUVECs measured by RT-qPCR. Bar plots: mean ± SD, two-tailed Student’s *t*-test, *n* = 4, **P < 0.01, ***P < 0.001.

Opposite to the primary role of BD in priming gene expression initially discovered in ES cells^3^, there are 45 genes among bdDEG were upregulated within 12 h course of VEGFA stimulation. In the following study, we set out to uncover this intrigue mechanism underlying the activation of these genes. Loss of the H3K27me3 repressive mark at bivalent genes was identified as a mechanism activating the BD-marked genes during the commitment of ES cell to tissue lineage. However, our ChIP-seq results showed that H3K27me3 mildly increased rather decreased at activated bdDEGs (middle panel, Fig. 2a). The chromatin signal of UTX and JMJD3, two demethylases specifically in charge with H3K27me3 demethylation, either maintained or reduced their chromatin occupancy at most bdDEG loci (Fig. 3a, b). These data suggested that VEGFA-dependent activation of bdDEGs did not share the same mechanism of H3K27me3 demethylation as was observed during stem cell lineage commitment.

*EZH1* recently has been shown to activate transcription of some genes, independent of H3K27me3^16,15,19^. VEGF increased the EZH1 protein level at 1 h although there were no significant changes in its mRNA level (Supplementary Fig. 3a, b). Thus, we hypothesized that upregulation of bdDEGs might be due to the transcriptional activation driven by EZH1 at these regions, and performed ChIP-seq to assess the EZH1 dynamics upon VEGF treatment at four time points of 0, 1, 4, and 12 h. The results demonstrated EZH1 significantly increased its binding at 1 and 4 h after stimulation at proximity of both activate and stable but not downregulated BD genes (Fig. 3c). Comparing to late-regulated genes, early-upregulated genes have more discernible increasement at 1 and 4 h after VEGF treatment (Supplementary Fig. 2). This elevated relocation to chromatin at 1 h was recapitulated by ChIP-quantitative polymerase chain reaction (qPCR) although not at 4 h (2.1-fold increase, *P* = 0.0046; Supplementary Fig. 3c). Suppressor Of Zeste12 Protein Homolog (SUZ12), an essential scaffolding component of PRC2 reported to associated with EZH1, also relocated to activated BD genes, which together with the evident of reduced EZH2 deposition suggested an atypical PRC2 complex mainly consisted of EZH1 and SUZ12, initially discovered within hematopoietic and heart cells, relocated to and elicited the bdDEG gene activation (Supplementary Fig. 3c–e)^15,16^.

To further interrogate if increased EZH1 relocation could in fact account for BD gene activation, we knocked down EZH1 with two independently designed *EZH1* short interfering RNAs (siRNAs) and then treated with VEGF for up to 4 h (Supplementary Fig. 4a). Reverse-transcription qPCR (RT-qPCR) demonstrated six tested BD genes selected from upregulated groups were activated by VEGF at 1 h, but abrogated after transfection of either EZH1 siRNA (Fig. 3d). The inhibition also lasted to 4 h especially on IGFBP3 and ADAMTS1, the expression of which kept to be upregulated up to 4 h. The ChIP-qPCR revealed the exist of BD at the same upregulated bdDEGs within human primary pulmonary artery (HPVECs), except *PROX1*, which is only expressed in venous and lymphatic endothelium (Supplementary Fig. 5a). We transfected the same *EZH1* siRNA to HPVEC, which markedly suppressed the activation of all five tested bdDEG with similar inhibitory extent in HUVEC (Supplementary Fig. 5b). Reversely, overexpressing EZH1 in HUVEC cells increased the expression of six BD genes along with upregulated deposition of H3K27me1, a putative substrate of EZH1 methyltransferase (Supplementary Fig. 6a). In contrast, a reduced H3K27me3 deposition was also observed in EZH1 overexpressed cells, suggesting a decreased H3K27me3 demethylases (Fig. 3a, b) rather an increased EZH1, were responsible for VEGF-altered H3K27me3 deposition near active bdDEG (Supplementary Fig. 6b).

### bdDEG activation depended on EZH1-mediated RNAPII pausing release

**Fig. 4:**
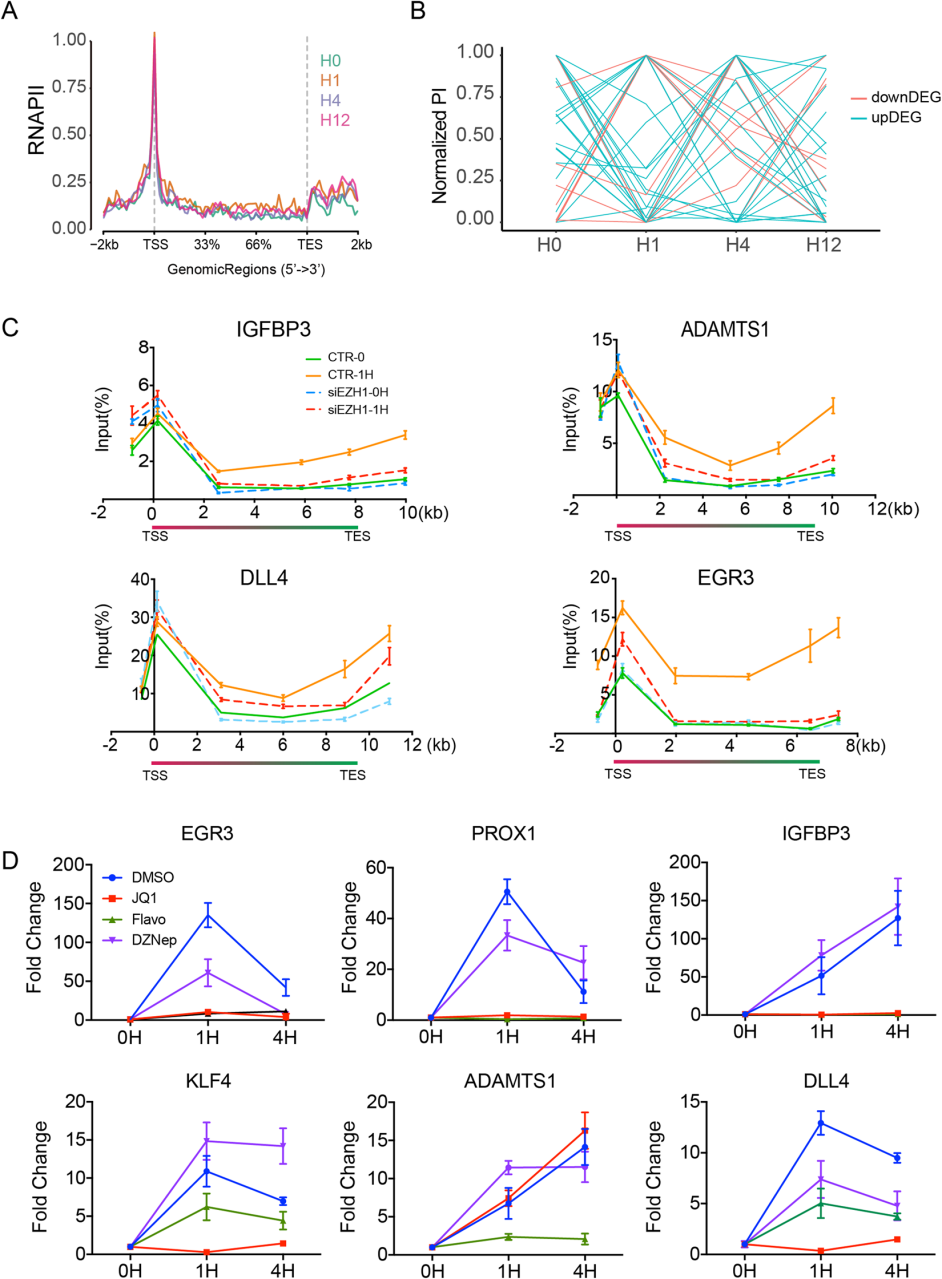
EZH1-induced RNAPII pausing release was required for the activation of bdDEG. **a** Metagene plot showing RNAPII occupancy of upregulated bdDEG at 0, 1, 4, or 12 h of VEGFA treatment. VEGFA treatment stimulated release of paused RNAPII at 1 h. **b** The RNAPII PI of downregulated bdDEG (red) and upregulated bdDEG (blue) at hour 0, 1, 4, and 12. **c** Effect of inhibition of RNAPII pausing release or PRC2 methyltransferase activity on VEGFA activation of bdDEGs, as measured by RT-qPCR. HUVECs were treated with VEGFA and/or small molecule inhibitors (JQ1, flavopiridol, and DZNep) or vehicle (DMSO). Inhibition of RNAPII pause release (JQ1 and flavopiridol) but not PRC2 methyltransferase activity (DZNep) suppressed VEGFA-driven transcriptional activation of the six tested bdDEGs. Plots show mean ± SD; *n* = 4, two-tailed Student’s *t*-test: *P < 0.05 compared to control at the same time point. **d** RNAPII pausing status with or without *EZH1* knockdown upon VEGF treatment for 0 or 1 h, as measured by RNAPII ChiP-qPCR. *EZH1* siRNA significantly abolished VEGF-induced RNAPII pausing release at four tested genes: *IGFBP3*, *ADAMTS1*, *DLL4*, and *EGR3*. Plots show mean ± SEM; *n* = 4.

After assembling at the promoter, at many genes, RNAPII initiates transcription and enters early elongation, and then pauses at 30–50 bp downstream of the TSS^20^. To complete the transcription, RNAPII need to be released from the pausing state. RNAPII pausing and pause release is rate-limiting for the transcription of many genes and is controlled by both positive and negative regulatory mechanisms^21^. Our previous studies reveal that VEGFA promotes RNAPII pausing release, which is essential for VEGFA-stimulated transcription and angiogenesis^11,20^. Notably, *EZH1* was reported to globally associate with H3K4me3 and release RNAPII pausing in myoblast cells^19^. Therefore, we further hypothesized that the activation of bdDEGs was executed by EZH1 and EZH1-mediated RNAPII pause release. To test this hypothesis, we first analyzed our previously published RNAPII ChIP-seq data^11^. Metagene plots of RNAPII signal at upregulated bdDEGs illustrated that most of these genes had paused RNAPII (Figs. 1a and 4a, Supplementary Fig. 4). After 1 h of VEGFA treatment, more RNAPII entered into the gene body illustrating RNAPII pausing release^20,22^. The pausing index (PI: the ratio of RNAPII signal near the TSS compared to within the gene body, see Methods section) is a widely used indicator of the extent of RNAPII pausing. Consistent with the metagene plot, PI of the majority of upregulated bdDEGs decreased markedly at 1 h (Fig. 4b).

To further confirm that EZH1 mediated the RNAPII pausing release at the upregulated bdDEG, we compared the RNAPII signals measured by RNAPII ChIP-qPCR at multiples sites across the mRNA transcription regions of four tested genes before and after EZH1 knockdown. Suppression of EZH1 attenuated the induction of RNAPII occupancy at transcriptional and transcriptional termination regions of four tested genes, illustrating a disabled RNAPII pausing release caused by EZH1 depletion (Fig. 4c). Furthermore, to confirm the role of RNAPII pause release in bdDEG activation, we blocked pausing release with two inhibitors JQ1 and flavopiridol, which inhibit the RNAPII release proteins BRD4 and CDK9, respectively. At six tested upregulated bdDEGs, both JQ1 and flavopiridol significantly dampened VEGFA-induced activation, with the exception of ADATMTS1, which was not affected by JQ1 (Fig. 4d). In the same experimental context, DZNep, a general histone methyltransferase inhibitor, had a much weaker effect (Fig. 4d). Thus, these results indicated that VEGFA activation of bdDEGs depends on EZH1-enhanced RNAPII pause release, rather the loss of H3K27me3.

### BD facilitated the silence of bdDEG though the recruitment of KDM5A

**Fig. 5:**
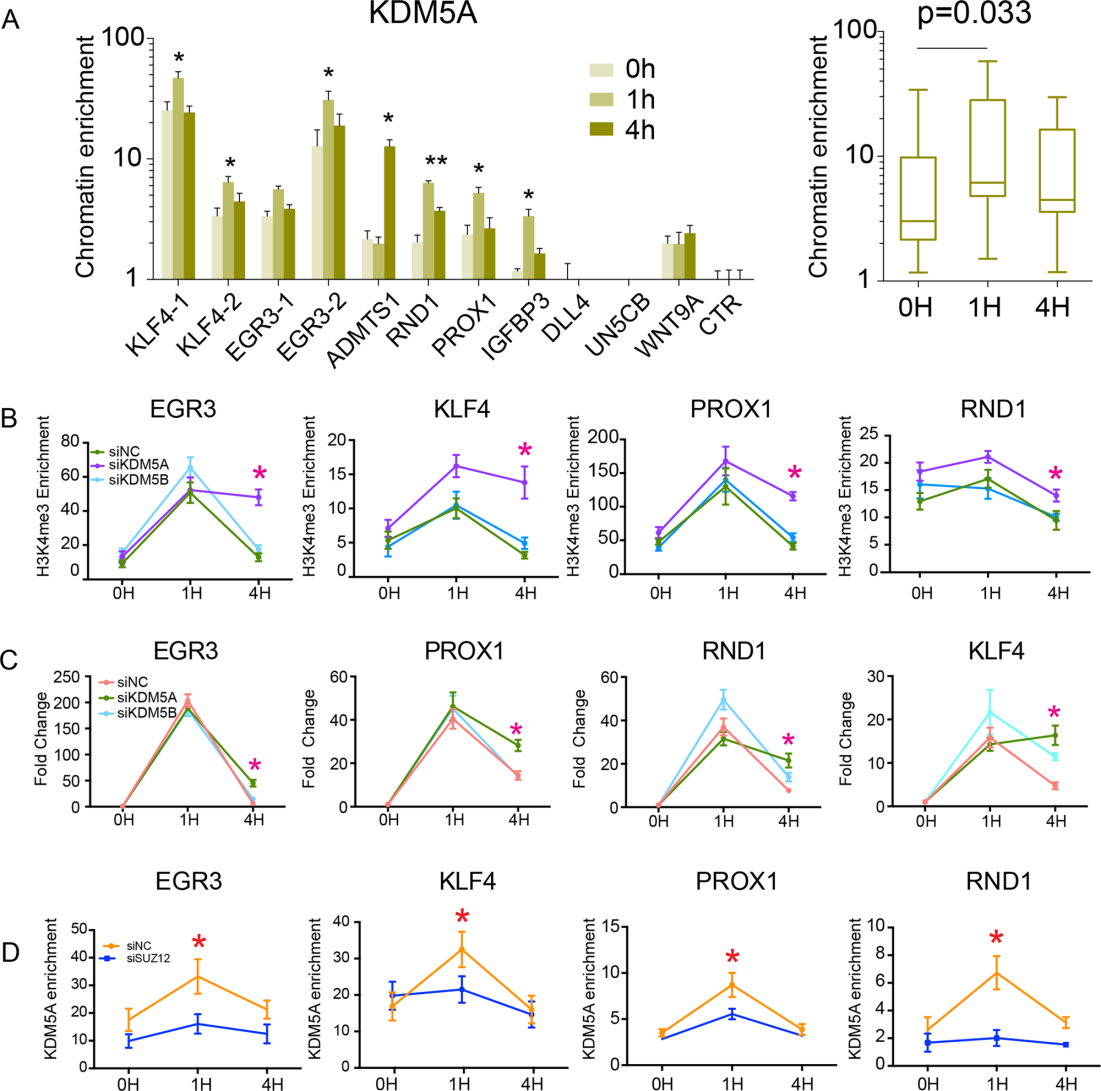
KDM5A facilitated the turning-off of bdDEG. **a** KDM5A occupancy of upregulated bdDEG promoters. VEGFA increased KDM5A binding near the TSS of upregulated bdDEG. The right box plot summarizes the change of in chromatin occupancy at each time point for all of the loci probed in the bar graphs to the left. Bars show mean ± SEM; *n* = 4, unpaired two-tailed Student’s *t*-test in left panel: *P < 0.05; **P < 0.01. Mann–Whitney *U* test in the right panel *P* < 0.05 indicated significant. **b** Effect of *KDM5A* or *KDM5B* inhibition on VEGFA activation of bdDEGs. HUVECs were treated with siRNA against *KDM5A* or *KDM5B*, or negative control siRNA (siNC) and then stimulated with VEGFA. Gene expression of four rapidly activated bdDEGs was measured at the indicated time points by RT-qPCR. *KDM5A* but not *KDM5B* siRNA sustained bdDEG expression at 4 h after VEGFA treatment, when expression of these genes has normally returned to baseline. Plots show mean ± SEM; *n* = 4, unpaired two-tailed Student’s *t*-test: *P < 0.05 compared to siNC at the same time point. **c** Effect of knockdown of *KDM5A* or *KDM5B* on H3K4me3 promoter signal of BD genes rapidly and transiently upregulated by VEGFA. Knockdown of *KDM5A* but not *KDM5B* significantly attenuated removal of H3K4me3 at four tested genes at H4. Data are plotted as the mean ± SEM; *n* = 4, unpaired two-tailed Student’s *t*-test: *P < 0.05. **d** Effect of *SUZ12* knockdown on KDM5A recruitment to the promoters of BD genes rapidly and transiently upregulated by VEGFA. Knockdown SUZ12 reduced KDM5A enrichment. Data are plotted as the mean ± SEM; *n* = 4, unpaired two-tailed Student’s *t*-test: *P < 0.05.

DEGs with bivalent promoters were most highly enriched for genes that were rapidly upregulated 1 h after VEGFA and then rapidly downregulated (Fig. 1d, Supplementary Table 2). Mirroring this expression time course, H3K4me3 promoter occupancy at early upregulated bdDEGs declined notably after 1 h (Figs. 1e and 2a). We hypothesized that H3K27me3 at these early and transiently upregulated bdDEG promoters functions to accelerate erasure of H3K4me3 and reduction of transcription. KDM5A, an H3K4me3 demethylase, was previously reported to physically interact with PRC2 (ref. ^6^) and therefore was a candidate that might link H3K27me3 at bdDEGs to H3K4me3 removal. By ChIP-qPCR, KDM5A occupancy of these bdDEG promoters dramatically increased at 1 h (Fig. 5a). Furthermore, *KDM5A* but not *KDM5B* siRNA suppressed the erasure of H3K4me3 and attenuated gene downregulation at 4 h (Fig. 5b, c, Supplementary Fig. 4a, b). Depletion of PRC2 by *SUZ12* siRNA reduced KDM5A occupancy at these promoters (Fig. 5d and Supplementary Fig. 4a, b), consistent with PRC2-dependent recruitment of KDM5A^6^. All these results suggest that H3K27me3 and KDM5A function together to accelerate the inactivation of rapidly upregulated bdDEGs by facilitating the removal of H3K4me3.

### BD governed endothelial cell migration and growth by modulating EGR3 expression

**Fig. 6:**
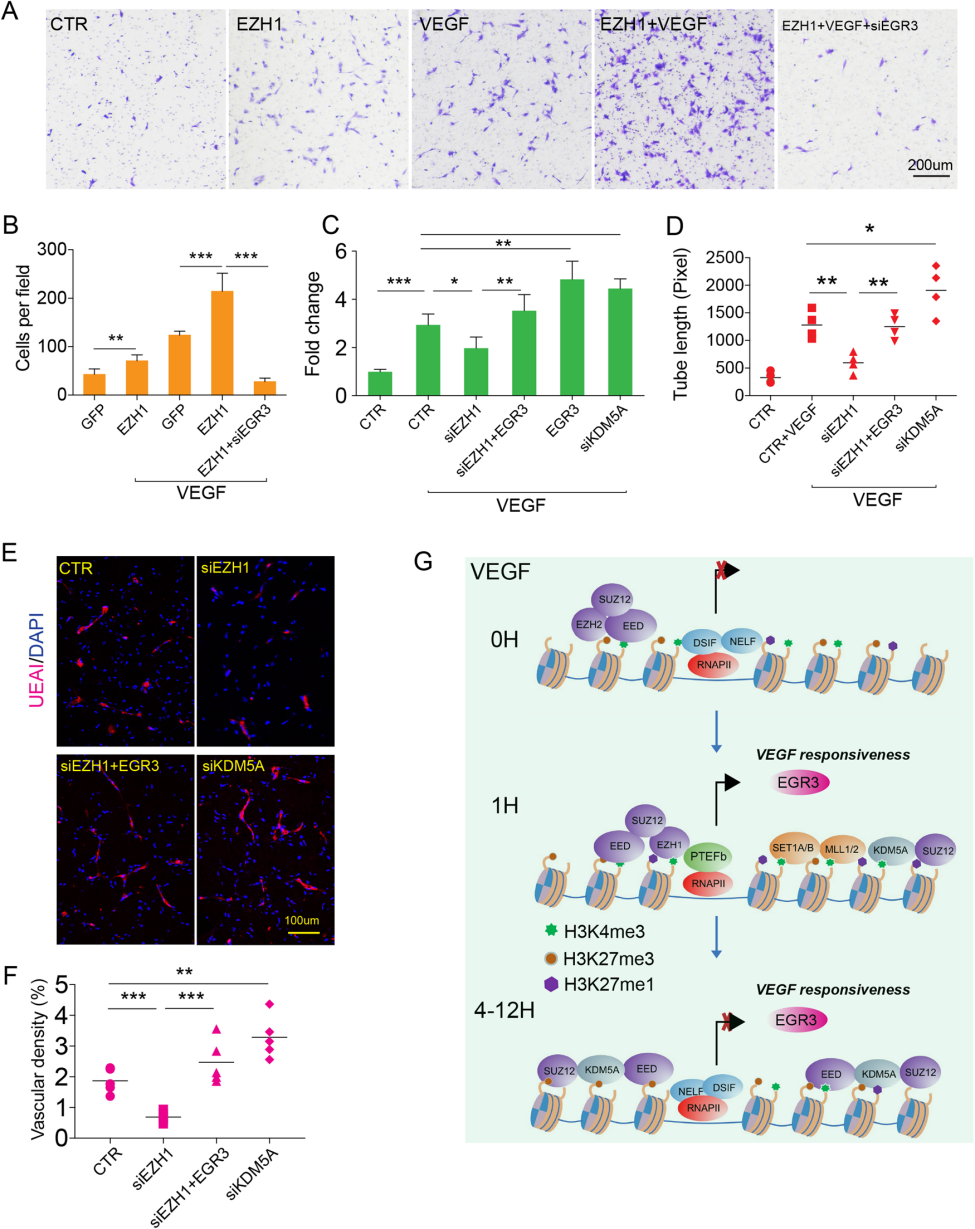
The effects of *EZH1*, *EGR3*, and KDM5A on neovascularization. **a**, **b** Transwell assay showing *EZH1* promoted HUVEC cell migration in culture conditions with or without VEGF, which was abolished by knocking down *EGR3*. **a** Migrated HUVECs on the transwell membrane revealed by crystal violet staining. **b** Calculation of migrated cells per field. Plots: mean ± SD, **P < 0.01; ***P < 0.001; *n* = 6, unpaired two-tailed Student’s *t*-test. **c** Proliferation of HUVECs measured by the CCK8 method. The suppression of *EZH1* abolished VEGF-induced HUVEC cell proliferation, which was reversed by addition of *EGR3*. Suppression of *KDM5A* enhanced cell proliferation. Bar plots: mean ± SD; *n* = 5, unpaired two-tailed Student’s *t*-test, ns no significance, *P < 0.01; **P < 0.01; ***P < 0.001. **d** Tube formation of HUVECs on Matrigel coat. *EZH1* siRNA dampened VEGF-induced tube formation of HUVECs, which was ameliorated by ectopic expression of *EGR3*; *KDM5A* suppression augmented VEGF-induced tube formation. Calculation of tube length in each tested group. Bar plots: mean ± SD, *n* = 4, unpaired two-tailed Student’s *t*-test, **P < 0.01, ***P < 0.001. **e**, **f** Matrigel assay evaluating the in vivo angiogenesis. **e** Representative images of neovasculature within transplanted Matrigel. UEAI-positive cells (magenta) indicating the wiring vasculature. **f** Dot plots of vascular density of UEAI-positive vasculature; mean ± SD, *n* = 5, unpaired two-tailed Student’s *t*-test, **P < 0.01, ***P < 0.001. **g** Mechanistic model of BD’s regulation of VEGFA responsiveness.

The preferential regulation of VEGF responsive and angiogenic gene expression at bivalent regions lead us to hypothesize BD may function in regulating angiogenesis. We tested this hypothesis by upregulating *EZH1* in HUVECs and examined its effect on endothelial cell migration, one of the symbolic features of angiogenesis. Upon VEGF stimulation, *EZH1* overexpression enhanced endothelial cell migration (*P* < 0.001, unpaired Student’s *t*-test, Fig. 6a). *EGR3*, a well-defined proangiogenic factor reported to enhance endothelial migration and proliferation, was induced by VEGF over a 100-fold through an EZH-controlled RNAPII pausing release^23,24^ (Fig. 1d, Supplementary Table 2). Thus, we anticipated that *EZH1*-induced HUVEC migration might be through the upregulation of *EGR3*. We knocked down *EGR3* by *EGR3*-specific siRNA that significantly attenuated *EZH1*’s augmentation in migration (Fig. 6a, b, Supplementary Fig. 7a). Noteworthy, *EZH1* also promoted the migration of HUVECs untreated with VEGF, which agreed with its function as a downstream effector of the VEGF pathway (Fig. 6a, b, Supplementary Fig. 7c, d). Conversely, knocking down *EZH1* dampened VEGF-induced HUVEC cell migration, which was restored by *EGR3* overexpression (Supplementary Figs. 6c, d and 7b). Depletion of *KDM5A* prolonged *EGR3* expression (Fig. 5c). Consistently, *KDM5A* siRNA significantly increased the HUVECs’ migratory ability (Supplementary Fig. 7a, b). Similar results were also observed in HPVECs, that HPVEC migration was promoted by *EZH1* and suppressed by *KDM5A*, which were both *EGR3* dependent (Supplementary Fig. 7e–h).

Neovascularization requires extensive cell growth^25^. We first assessed the effects of *EZH1* and *KDM5A* on the growth rate of HUVECs by measuring cell viability with CCK8 (Methods section). VEGF induced significantly increased cell growth after three days of administration (Fig. 6c). Knocking down *EZH1* mildly but significantly attenuated this induction (Fig. 6c). Recovering *EGR3* in the *EZH1*-depleted cells completely restored the growth of the HUVECs, which agreed with the pro-proliferation function of *EGR3* shown by upregulating *EGR3* alone^24^. Likewise, using *KDM5A* siRNA to extend *EGR3* expression increased cellular proliferation (Fig. 6c). Conversely, ectopic expression of *EZH1* enhanced VEGF-induced cell growth, which was abolished by further adding *EGR3* siRNA (Supplementary Fig. 7i).

Angiogenic endothelial cells are able to form a network on the surface of extracellular matrix such as Matrigel, which is an indicator of angiogenic properties. We deployed the tube formation assay to evaluate the effects of EZH1, EGR3, and KDM5A on the endothelial network formation. *EZH1* siRNA dampened the formation of HUVEC tubes as indicated by a shorter tube length within the measured regions (Fig. 6d, Supplementary Fig. 8a). Further upregulation of *EGR3* rescued the impaired tube formation capacity upon the depletion of *EZH1* (Fig. 6d, Supplementary Fig. 8a). *KDM5A* siRNA augmented the tube formation of HUVECs primed with VEGF stimulation (Fig. 6d, Supplementary Fig. 8a). With a similar effect on endothelial cell proliferation and migration, ectopic expression of *EZH1* promoted HUVEC tube formation, which depended on the upregulation of *EGR3* (Supplementary Fig. 8b, c). Together, these lines of evidence demonstrate that the BD in endothelial cells controls VEGF-induced in vitro angiogenesis by modulating expression of its target gene EGR3.

### BD governed in vivo angiogenesis

To further probe the regulation of BD on in vivo angiogenesis, we carried out a Matrigel plug transplantation assay in immunodeficient mice (Methods section). Same as in the in vitro experiment, we first manipulated *EZH1*, *KDM5A*, and *EGR3* levels in the HUVECs by using siRNA or lentivirus and then transplanted them to the rodent subcutis after mixing them with Matrigel and MSCs that supported the formation of functional vasculature. This assay demonstrated that suppression of *EZH1* in endothelial cells impaired the neovascularization within the Matrigel, whereas suppression of *KDM5A* enhanced it (Fig. 6e, f). Restoring the *EGR3* in *EZH1*-depleted cells also rescued their angiogenic capacity (*P* < 0.001, unpaired Student’s *t*-test, Fig. 6e, f). A similar observation from both in vitro and in vivo angiogenesis assays concluded that the BD is able to control angiogenesis not only in vitro but in vivo.

## Discussion

This study reveals a previously unrecognized role and mechanism of BDs in regulating signal-responsive genes. Before VEGFA stimulation of endothelial cells, EZH2-containing PRC2 occupies bdDEGs and suppresses gene expression through RNAPII pausing. After VEGFA treatment, EZH1 rapidly replaces EZH2 and releases paused RNAPII with increasing H3K27me1 and H3K4me3 deposition possibly through the recruitment of H3K4me3 methyltransferases. Together, these actions rapidly increase gene transcription. On the other hand, the existence of H3K27me3 at the BD facilitates later inactivation of early-responsive bdDEGs at 4 and 12 h, H3K27me3 recruits KDM5A to these bdDEGs, resulting in removal of H3K4me3 and transcriptional inactivation. The two faces of BD controlling the expression of VEGF-responsive genes, especially EGR3, play a pivotal role in the activation and inactivation of in vitro and in vivo angiogenesis (Fig. 6g).

### Bivalency remains at BDs in endothelial cells

The functions of BDs have been well described during ES cell differentiation, but not clearly illustrated in the committed cells. Here, we revealed that bivalency bears dual roles in regulating VEGFA-induced gene expression but the interpretation of bivalency is entirely different. In the activation phase of VEGFA stimulation, rather to remove H3K27me3 for activation of gene expression, the BD was activated through a mechanism in which EZH1 released the paused RNAPII at these regions^19^. Although both *EZH1* and *EZH2* trimethylate histone H3 on lysine 27, *EZH1* has been shown to positively regulate gene expression in multiple tissues. In myoblasts, *EZH1* globally associated with H3K4me3 and RNAPII, promotes RNAPII elongation, and activates genes that govern the differentiation into skeletal muscle cells^19^. An *EZH1/EZH2* switch mediated by *GATA1/GATA2* transition was discovered in erythroid progenitor cells^26^. In the neonatal heart, EZH1 also positively regulated genes related to heart repair^16^. In line with these discoveries, we found in the blood vessels, EZH1 was redirected to the BD and positively regulated endothelial genes there in the context of VEGFA stimulation.

The bivalent nature of BD domains facilitated timely inactivation of VEGFA-upregulated genes: H3K27me3 at these domains was bound by PRC2, leading to KDM5A recruitment, H3K4me3 reduction, and gene silencing. Unlike the complete erasure of H3K4me3 during ES differentiation, the reduction of H3K4me3 at endothelial cell BDs was incomplete, leaving the BD competent to properly respond to subsequent VEGFA stimulation. The mechanism that limits KDM5A demethylation of these BDs is not known and will be an interesting area for future exploration.

### Potential functions of BDs in adult tissue

BDs are not restricted to multipotent stem cells; they are also found in differentiated, mature cells. Many tissues have been found to have over 1000 BDs^4^. The function of BDs in mature cells has not been elucidated. Here, we showed that BDs in endothelial cells are able to regulate VEGFA-stimulated transcription and angiogenesis. This further emphasizes there are critical functions of BD in adult tissues. Clarifying BD’s function and regulation in other tissues or other important biological processes will become an interesting and important area for future exploration.

## Materials and methods

### Cell culture

For routine culture, HUVECs (CC-2519, Lonza) and human pulmonary artery endothelial cell (HPAEC, CC-2530, Lonza) between three to seven passages were maintained in endothelial growth medium medium 2 (EGM2, cc-3162, Lonza) with all the growth factor supplied. For VEGFA stimulation experiments, HUVECs were cultured overnight in basal endothelial cell growth medium 2 medium (EBM2, CC-4176 Lonza) with 1% fetal bovine serum (FBS). A total amount of 50 ng/ml VEGFA was then added, and cells were collected at 0, 1, 4, and 12 h for ChIP-qPCR, ChIP-seq, RNA-seq, and RT-qPCR. Where indicated, flavopiridol (100 nM), JQ1 (500 nM), or DZNep (500 nM) were added 1 h before VEGFA stimulation.

Human MSCs (hMSCs) were purchased from Lonza (Catalog #: PT-2501) and cultured in MSCBM^TM^ MSC basal medium (PT-3238, Lonza).

siRNAs were synthesized at GenePharma with all sequences listed in the Supplementary Table 3. A total amount of 10 ng/ml siRNA were transfected with Lipofectamine® RNAiMAX regent manufactured by Thermo Fisher Scientific. The serum starvation was performed one day after siRNA transfection.

### Mice

All mice experiments were performed under protocols approved by the Institutional Animal Care and Use Committee of Shanghai Jiao Tong University.

### Matrigel plug assay

Transplanted Matrigel plug assays were performed as described previously^11^. Briefly, 1 × 10^6^ HUVECs at 4–6 passage and 2 × 10^6^ human MSCs were mixed with 200 µl ice cold Matrigel (Corning, 356237) and injected into the subcutis of 6–8-week-old male nude mice (Si Lai Ke Experimental LLC, China) that were randomly subjected to different treatments after excluding the unhealthy entities. Five mice were assigned for each group. The Matrigel plugs were dissected out and embedded in paraffin. Ulex Europaeus Agglutinin I (UEAI, 1:200, Vector Labs), a lectin that recognizes human but not mouse endothelial cells, was used to reveal the vascular network formed by the transplanted HUVECs. Images were acquired with a Nikon A1Si confocal microscope, and UEAI positive vasculature was measured using ImageJ.

### Western blot

Total proteins were extracted from 2–5 × 10^6^ HUVECs by using RIPA buffer (2.5 mM Tris-HCl PH7.4, 150 mM NaCl, 1% NP40, 1% sodium dexycholate, 0.1% sodium dodecyl sulfate (SDS), and protease inhibitor cocktails). The protein concentration was measured with BCA Protein Assay (23225, Pierce). Equal amounts of protein were loaded onto each well, separated on 10% SDS–polyacrylamide gel electrophoresis gel, and transferred to immobilon-P PVDF membrane (IPVH000010, Millipore). Primary antibodies (Supplementary Table 4) were used to specifically probe the tested proteins. The protein bands were visualized with Immobilon Western Chemiluminescent HRP substrate (WBKLS0500, Millipore) and imaged on Amersham Imager 600.

### RT-PCR

A total of 1 × 10^6^ HUVECs were collected with cell lysis buffer RLT with 1% of 2-mercaptoemethanol. Total RNA was purified using the RNeasy Mini Kit with on-column DNase digestion. RNA quantity and quality were assessed by NanoDrop Spectrophotometer (Thermo Fisher). RNA samples with A260/A280 ratio > 1.8 were saved for RT-qPCR or mRNA-seq.

For RT-qPCR, first strand cDNA was synthesized from 1 µg total RNA with the SuperScript III First-strand synthesis System (Thermo Fisher). RT-PCR tests were carried out with PowerUp SYBR^TM^ Green Master Mix (Thermo Fisher) and detected by StepOnePlus Real-Time PCR systems (Thermo Fisher).

### ChIP-qPCR and ChIP-seq

ChIP was performed as described previously with minor optimization under the ENCODE guideline^27^. Approximately 2–10 × 10^7^ HUVECs after treatment with VEGFA for 0, 1, 4, or 12 h were crosslinked with 1% formaldehyde for 10 min at room temperature. After neutralizing with 0.125 M glycine for 5 min, the nuclei were extracted with 5 ml hypotonic buffer (20 mM HEPES pH 7.5, 10 mM KCl, 1 mM EDTA, 0.2% NP40, 10% glycerol, 1 × Protease Inhibitor Cocktail (PI; Roche), and then suspended in the sonication buffer (20 mM Tris Cl pH 8.0, 2 mM EDTA, 150 mM NaCl, 1% NP40, 0.1% SDS, and 1 × protease inhibitor). Chromatin were sheared by sonication (Bioruptor Plus, Diagenode: 30 s per cycle for 30 cycles). The sheared chromatin was precleared with 30 µl protein G Dynabeads (ThermoFisher Scientific) for 1 h, incubated with 5–10 µg ChIP antibody (Supplementary Table 4) at 4 °C overnight and then pulled down by Protein G Dynabeads (ThermoFisher) for 4 h at 4 °C. The beads with ChIPed DNA were rinsed three to five times with RIPA buffer (50 mM HEPES pH 8.0, 1 mM EDTA, 1% NP40, 0.7% sodium deoxycholate, 1% TritonX-100, 0.5 M LiCl, and 1× protease inhibitor) and then decrosslinked in SDS buffer (50 mM Tris-Cl pH 8.0, and 1% SDS) at 65 °C for 12–16 h. The reversely crosslinked DNA was purified with QIAquick PCR purification columns after digestion with RNase A and proteinase for 1 h each.

The purified DNA for ChIP-qPCR was directly detected with PowerUp SYBR^TM^ Green Master Mix (Thermo Fisher). The primers for each tested site are listed in Supplementary Table 3. The chromatin occupancy was calculated with the following formula: chromatin occupancy = 2 ^ [△Ct of test site (ChIP-input)-△Ct of control site (ChIP-input)].

For ChIP-seq, ChIP DNA was converted to Illumina sequencing libraries using the NEBNext® ChIP-seq Library Prep Master Mix Set (NEB) by following the manufacturer’s instructions. Size selection of DNA libraries after adding the sequencing adaptors was performed on agarose gels rather than with beads. The barcoding primers were custom prepared (Supplementary Table 3). The quality of the constructed libraries was evaluated by Tape station 2200 (Agilent). To obtain optimized cluster density in the flow cells, the barcoded DNA was quantified using the qPCR NGS library quantification kit (Illumina). Finally, 50 nt single-end reads were produced from Illumina HiSeq2000 or 2500 platforms.

### RNA-seq

RNA-seq was performed as described^11^. In brief, 5 µg total RNA from HUVECs was purified with the Dynabeads mRNA DIRECT Purification Kit (Thermo Fisher) following the manufacturer’s instructions. Oligo-dT beads were applied twice to purify the polyadenylated RNA in order to minimize the rRNA contamination. The libraries were constructed using Scriptseq V2 RNA-Seq Library Preparation Kit (Illumina). We used custom primers in the last step of PCR to add barcodes to the library DNA. The Illumina HiSeq 2500 platform was deployed to yield 50 nt pair-end reads. Two biological replicates at each time point of VEGFA treatment were sequenced.

### ChIP-seq

Sequencing reads were aligned to human genome hg19 using Bowtie2 with the default option. Duplicates were marked by Picard. Low quality reads having a mapping quality <15 were abandoned. MACS2 was applied to call peaks from aligned BAM files in the default setting. Peaks falling into blacklist regions (https://sites.google.com/site/anshulkundaje/projects/blacklists) were removed. To visualize histone signals at BD regions, the ngs.plot.r in ngsplot package (https://github.com/shenlab-sinai/ngsplot) was used (-G hg19–R genebody–L 5000–GO total–SC global).

### PI calculation

PI was calculated as described^12^ based on the RNAPII ChIP-seq. The RNAPII signal was first normalized by an input signal at the same region. The ChIP signal at the TSS (−50 to +300 bp) and gene body region (−300 of TSS to +2 kb of transcriptional end site, TES) was calculated using a home-written software package of PIC (https://github.com/binglab-SJTU/PIC) and the formula: PI = (normalized signal at TSS/length of TSS)/(normalized signal at gene body/length of gene body).

### *K*-means cluster

ChIP-seq signals of each histone modification (H3K4me2, H3K4me3, H3K27ac, and H3K27me3) near the TSS (TSS ± 1 kb) were calculated by the PIC package and then normalized to the signal of input. The number of clusters was estimated by the silhouette method and then grouped by *K*-means clustering. Fisher’s exact test was used to evaluate the enrichment of DEGs and bdDEGs in each TSS cluster.

### RNA-seq analysis

RNA-seq reads were aligned to human genome hg19 using Tophat2 (23). Cuffquant (24) was used to quantify the gene abundances with options ‘--multi-read-correct --frag-bias-correct’. Differential gene expression was assessed with CuffDiff (24) with options ‘--library-norm-method geometric --dispersion-method pooled --compatible-hits-norm --multi-read-correct --min-alignment-count 10 --FDR 0.1 --frag-bias-correct’. Genes with fold change ≥ 2 and FDR ≤ 0.1 at hour 1, 4, or 12 in pairwise comparison to hour 0 were selected as DEGs. A total of 901 DEGs, including 37 polyadenylated lncRNAs, were used for analysis in this study.

### Tube formation assay

The tube formation assay was performed as described^28^. Briefly, 24 well plates were coated with 300 µl of ice-cold Matrigel (Corning, 356237, 1:2 dilution with 200 µl EGM2 medium). After 1 h of gelatinization at 37˚C within a cell culture incubator, 1–5 × 10^5^ HUVECs treated with siRNA or infected with 1–3 MOI of lentivirus were plated on the gel surface with or without incubation of 30 ng/ml VEGFA. The endothelial tubes formed between 6–16 h and were imaged by a Nikon SMZ800N microscope. Total tube length was measured with ImageJ software from the acquired pictures.

### In vitro cell proliferation

HUVECs were transfected with 10 nm/ml of EZH1 or KDM5A siRNA or infected with EZH1 or EGR3 lentivirus (1–3 MOI) for 24 h, reseeded to 96 well plates (1000–3000 cells/well), and treated with 30 ng/ml of VEGFA for 72 h. The cells’ viability was measured using a Cell Counting Kit-8 (CCK8, Beyotime #C0042) following the manufacturer’s instructions. The OD_450_ value was quantified with an ELISA reader (BioTek) and converted to cell numbers using a standard curve drawn with a series of increasing cell numbers.

### Transwell migration assay

Transwell assays were carried out as previously described^28^. HUVECs and HPVECs were transfected with EZH1, KDM5A, or EGR3 siRNA or infected with EZH1, EGR3 lentivirus (1–3 MOI) for 48 h, trypsinized into single cells, and resuspended in migration medium (EBM2 + 1% FBS). Transwell chambers (Corning 3422, pore size 8 µm) were assembled in 24 well plates. Then, 1 × 10^5^ HUVECs or HPVECs in 100 µl of migration medium were added to the upper side of the transwell chamber, and 600 µl of migration medium with or without 30 ng/ml VEGF was added to the lower well to induce migration.

## Supplementary information

Supplemental figure 1

Supplemental figure 2

Supplemental figure 3

Supplemental figure 4

Supplemental figure 5

Supplemental figure 6

Supplemental figure 7

Supplemental figure 8

Table1

Table2

Table3

Table4

Supplemental Figure Legends

## Data Availability

All raw and processed NGS data are available in GEO 109626 and 139077 with token of ‘gfyduwmkhbqplop’ for reviewer.
