# Supplementary figures and images for: Two faces of bivalent domain regulate VEGFA responsiveness and angiogenesis
Source: Cell Death Dis. 2020 Jan 30;11(1):75. doi: 10.1038/s41419-020-2228-3 (PMC6992747; doi:10.1038/s41419-020-2228-3)

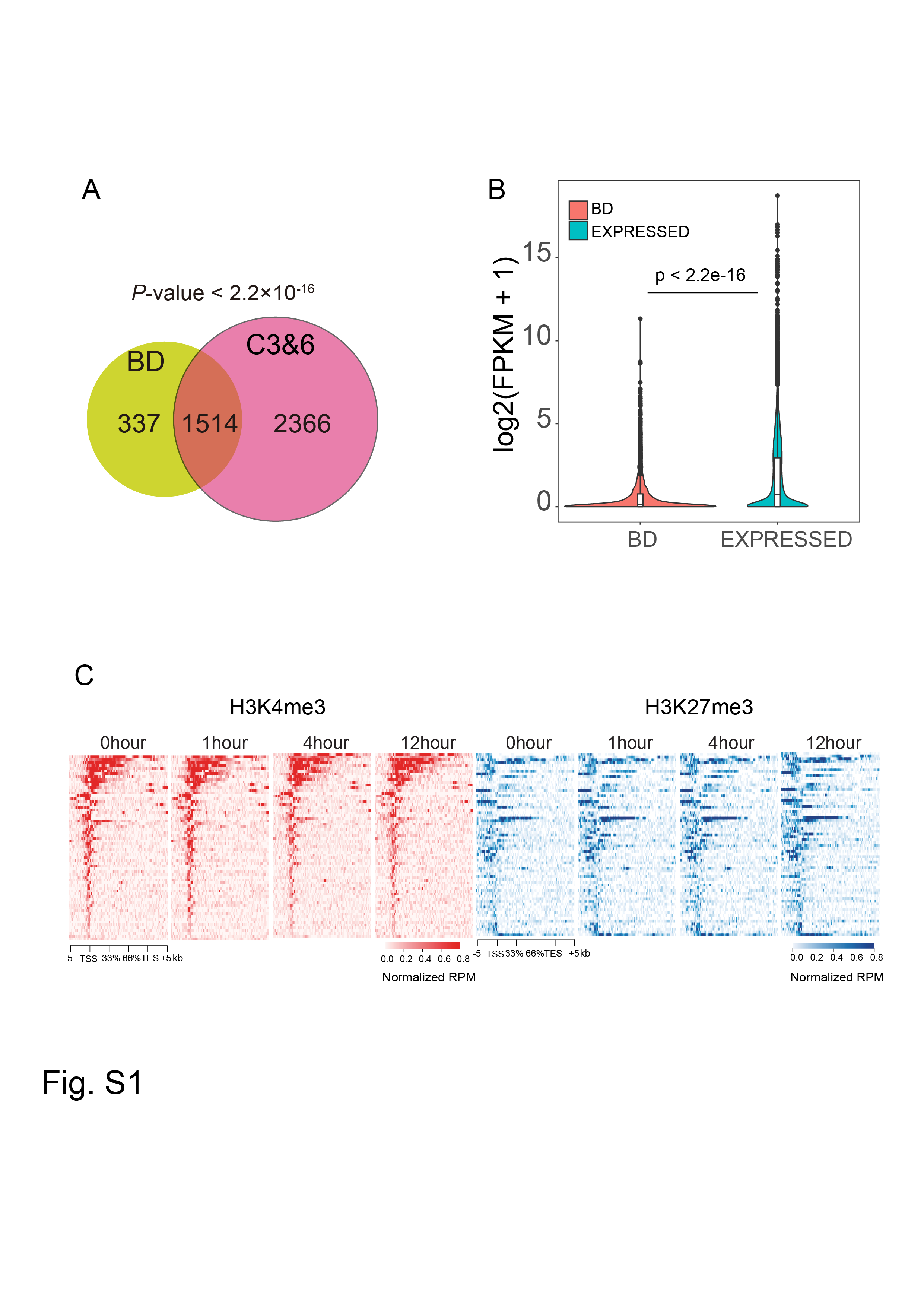

Supplement: Supplementary file 1 — Supplemental figure 1 [file 41419_2020_2228_MOESM1_ESM.png]

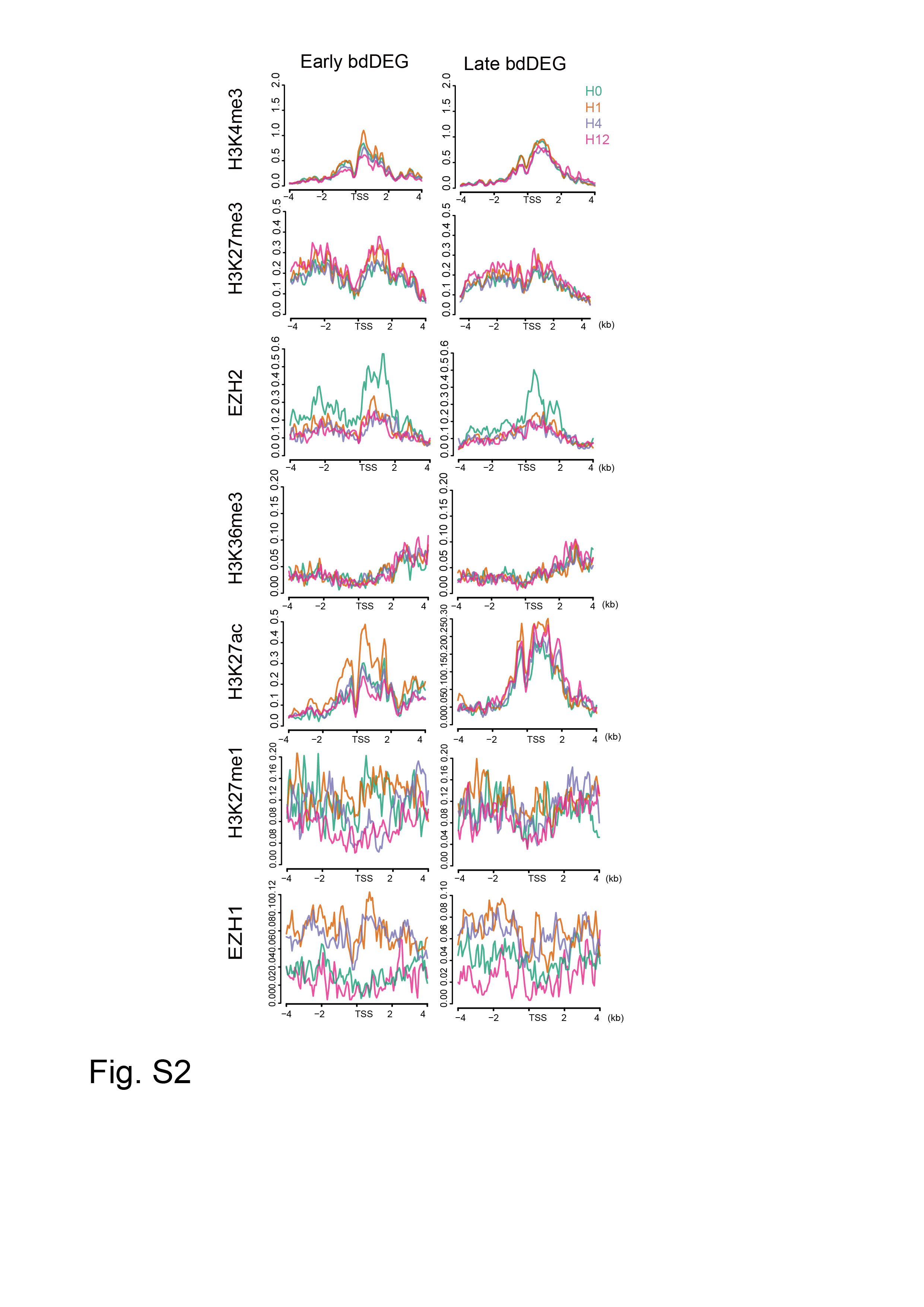

Supplement: Supplementary file 2 — Supplemental figure 2 [file 41419_2020_2228_MOESM2_ESM.png]

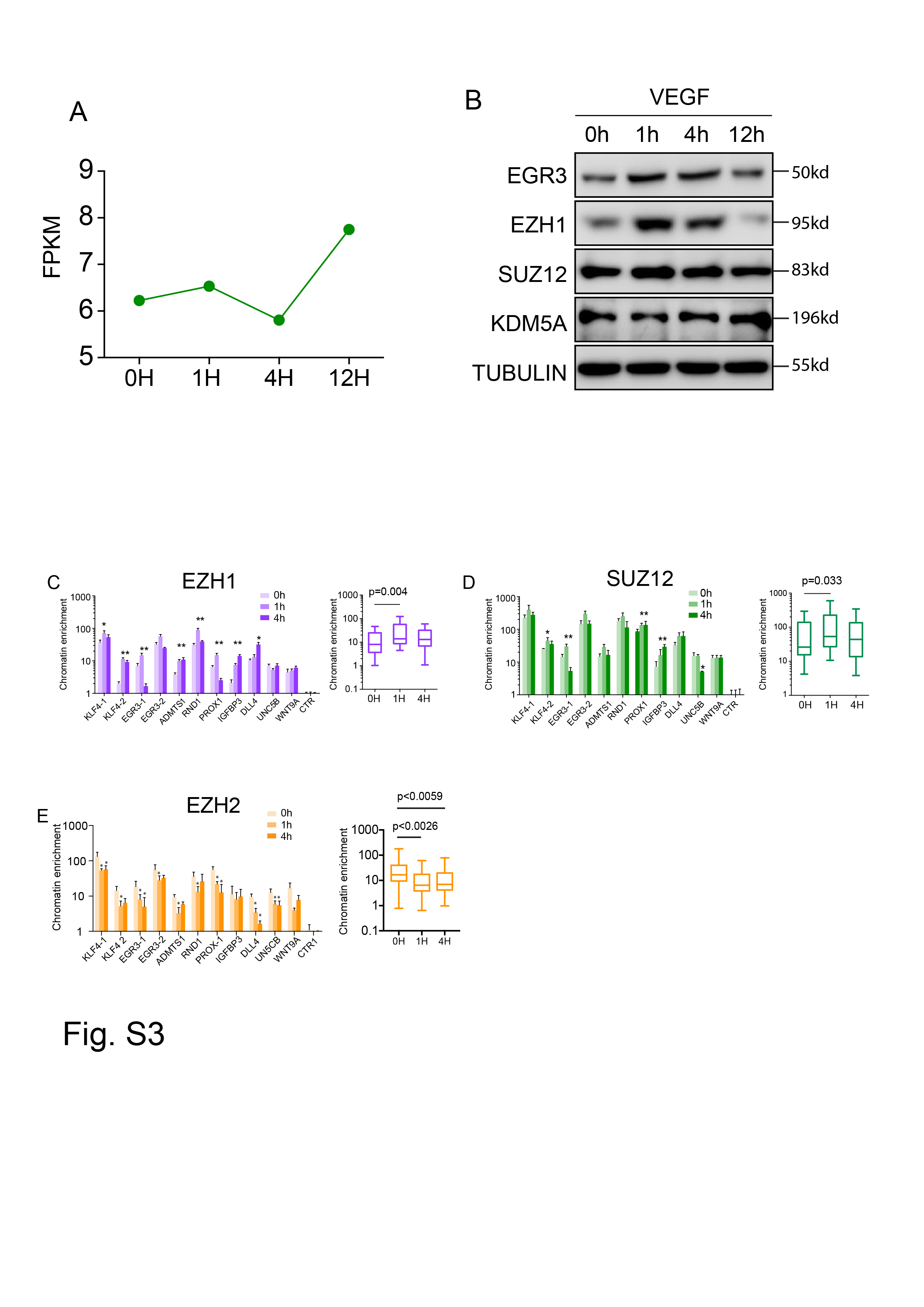

Supplement: Supplementary file 3 — Supplemental figure 3 [file 41419_2020_2228_MOESM3_ESM.png]

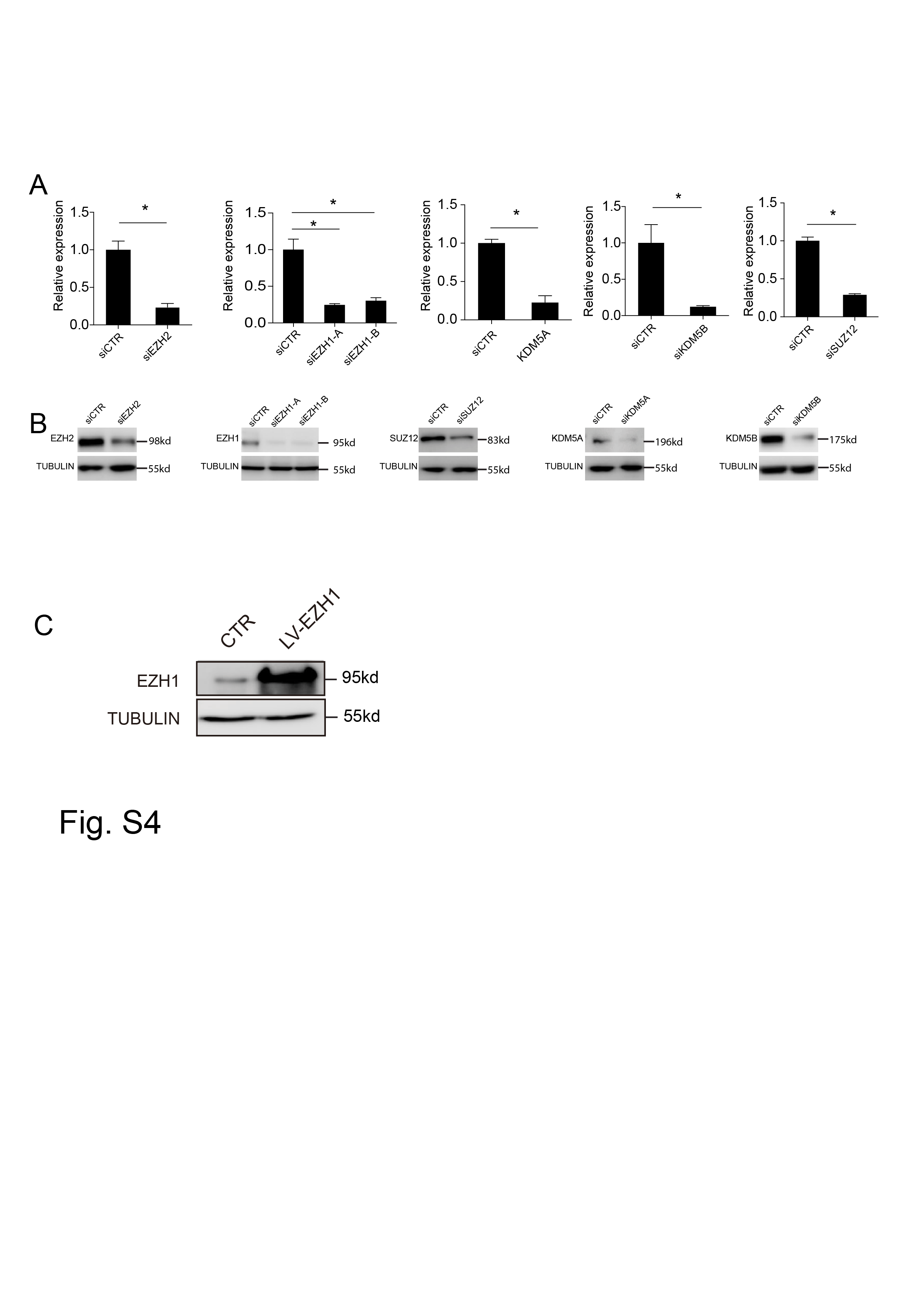

Supplement: Supplementary file 4 — Supplemental figure 4 [file 41419_2020_2228_MOESM4_ESM.png]

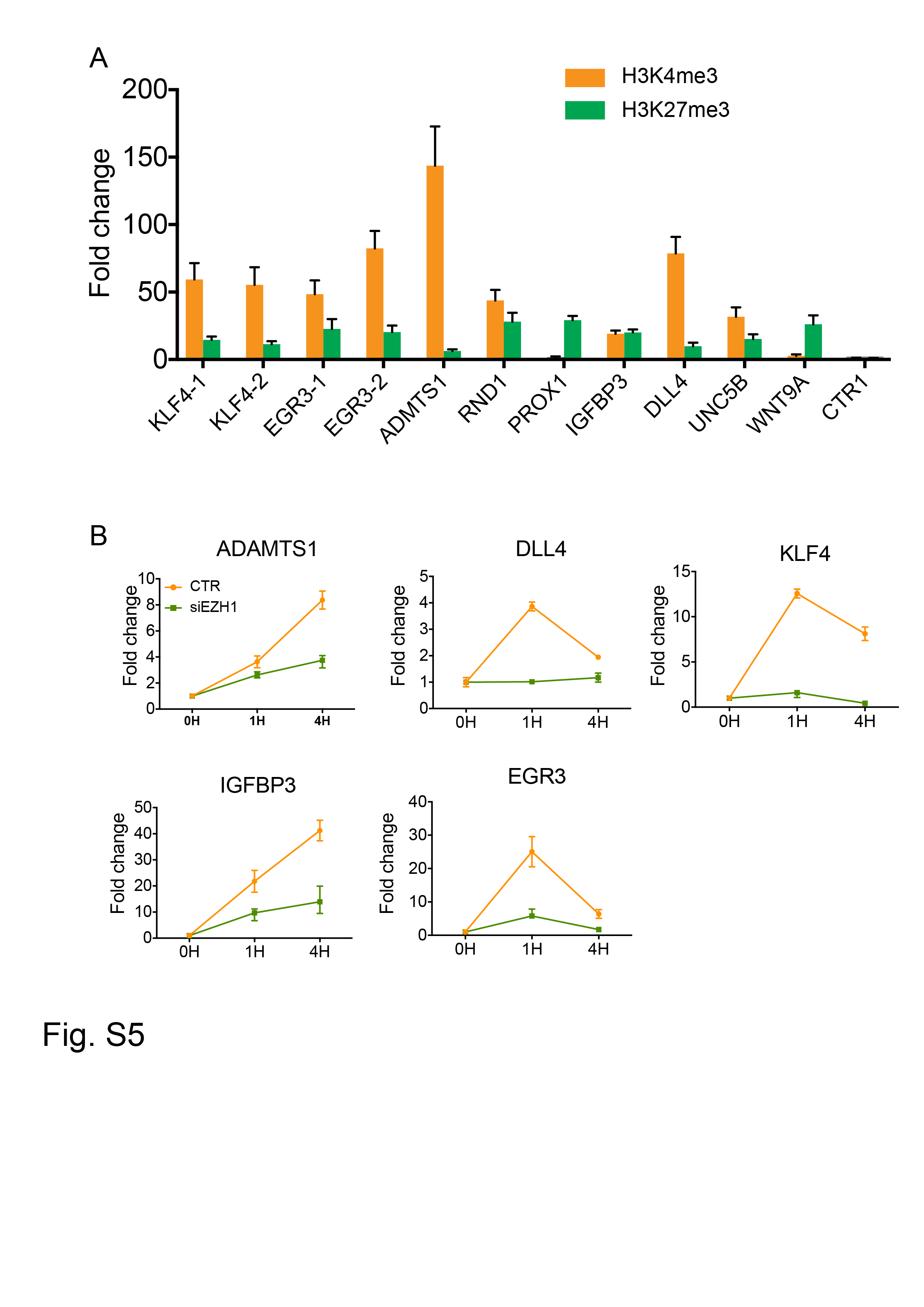

Supplement: Supplementary file 5 — Supplemental figure 5 [file 41419_2020_2228_MOESM5_ESM.png]

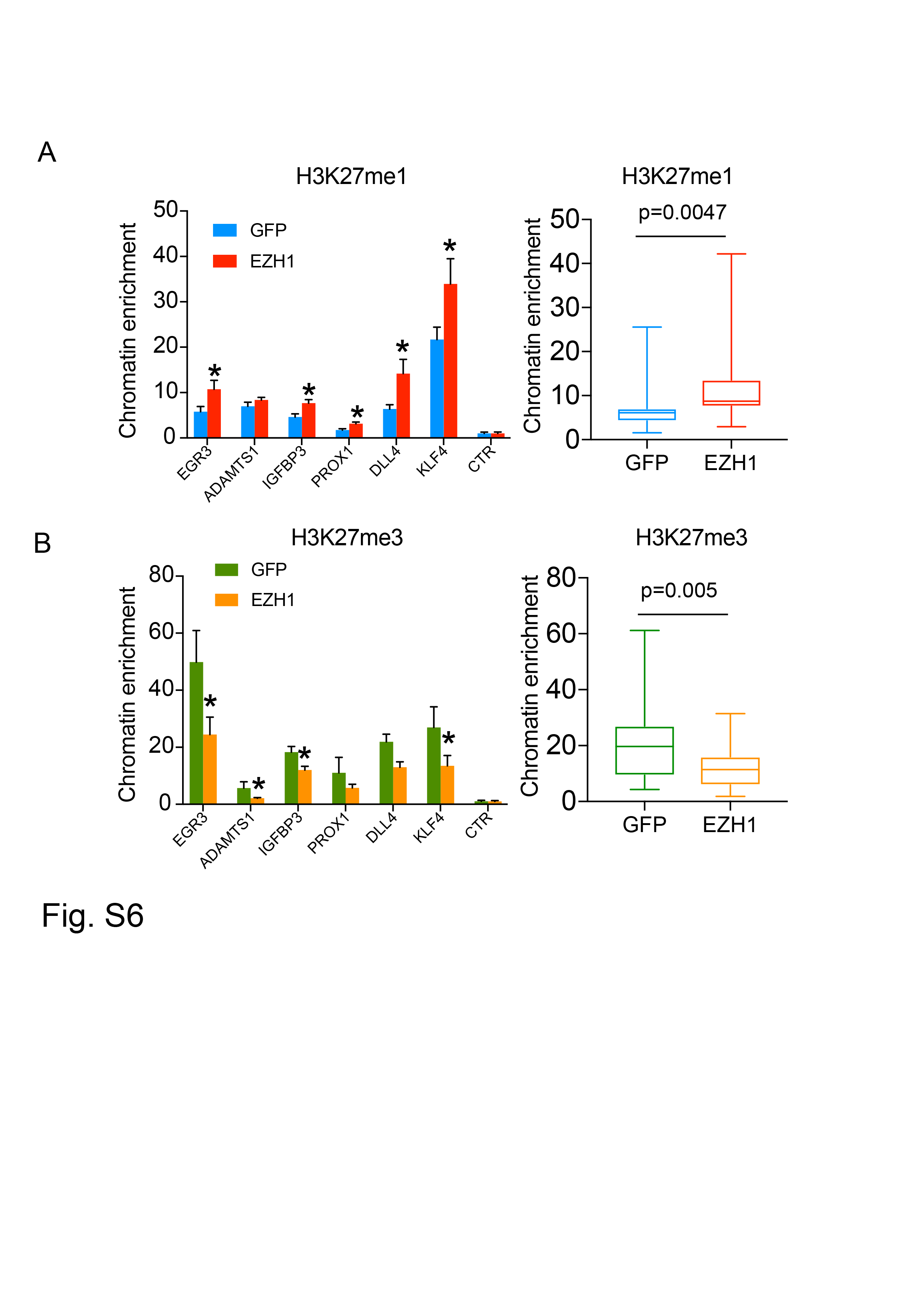

Supplement: Supplementary file 6 — Supplemental figure 6 [file 41419_2020_2228_MOESM6_ESM.png]

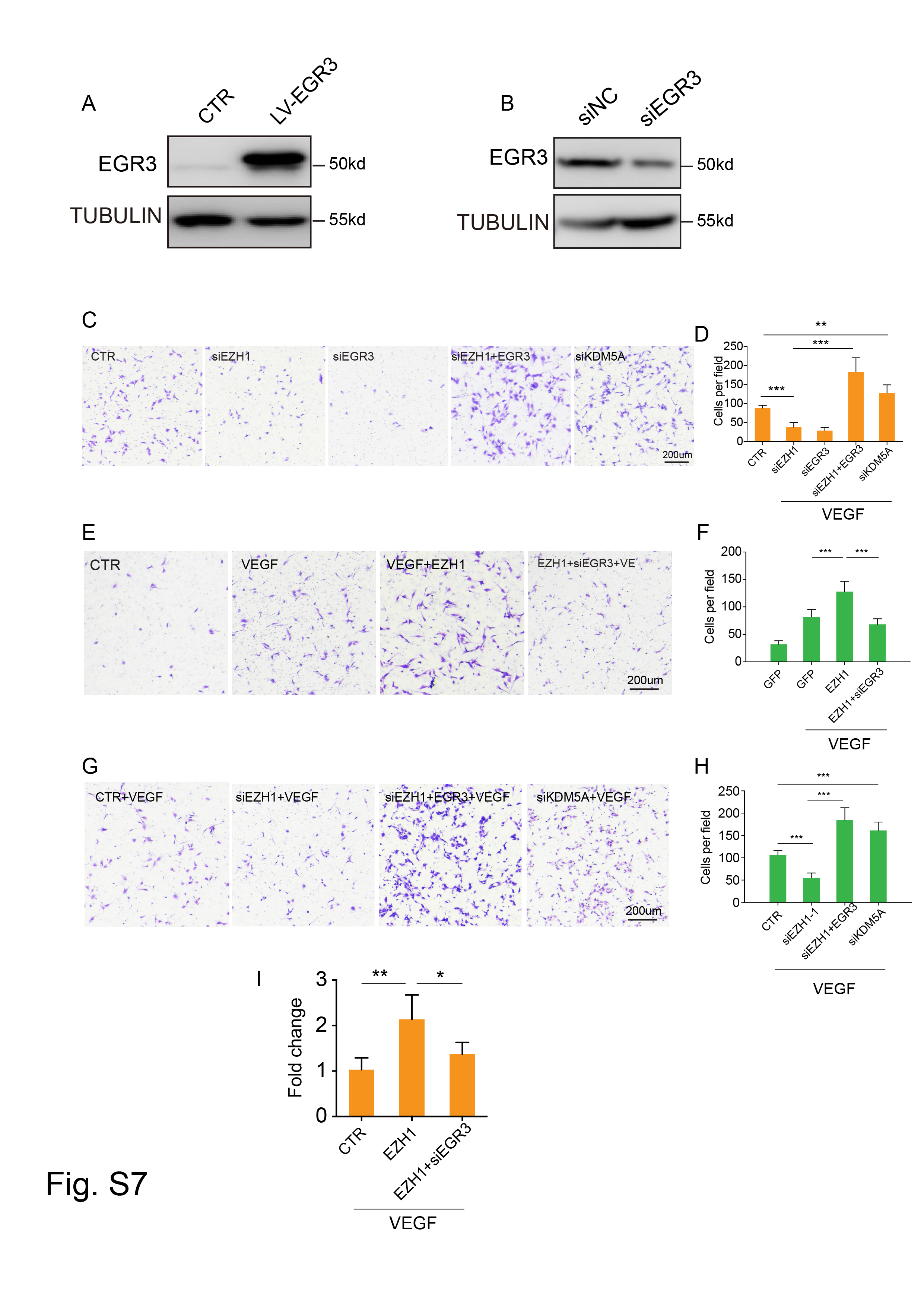

Supplement: Supplementary file 7 — Supplemental figure 7 [file 41419_2020_2228_MOESM7_ESM.png]

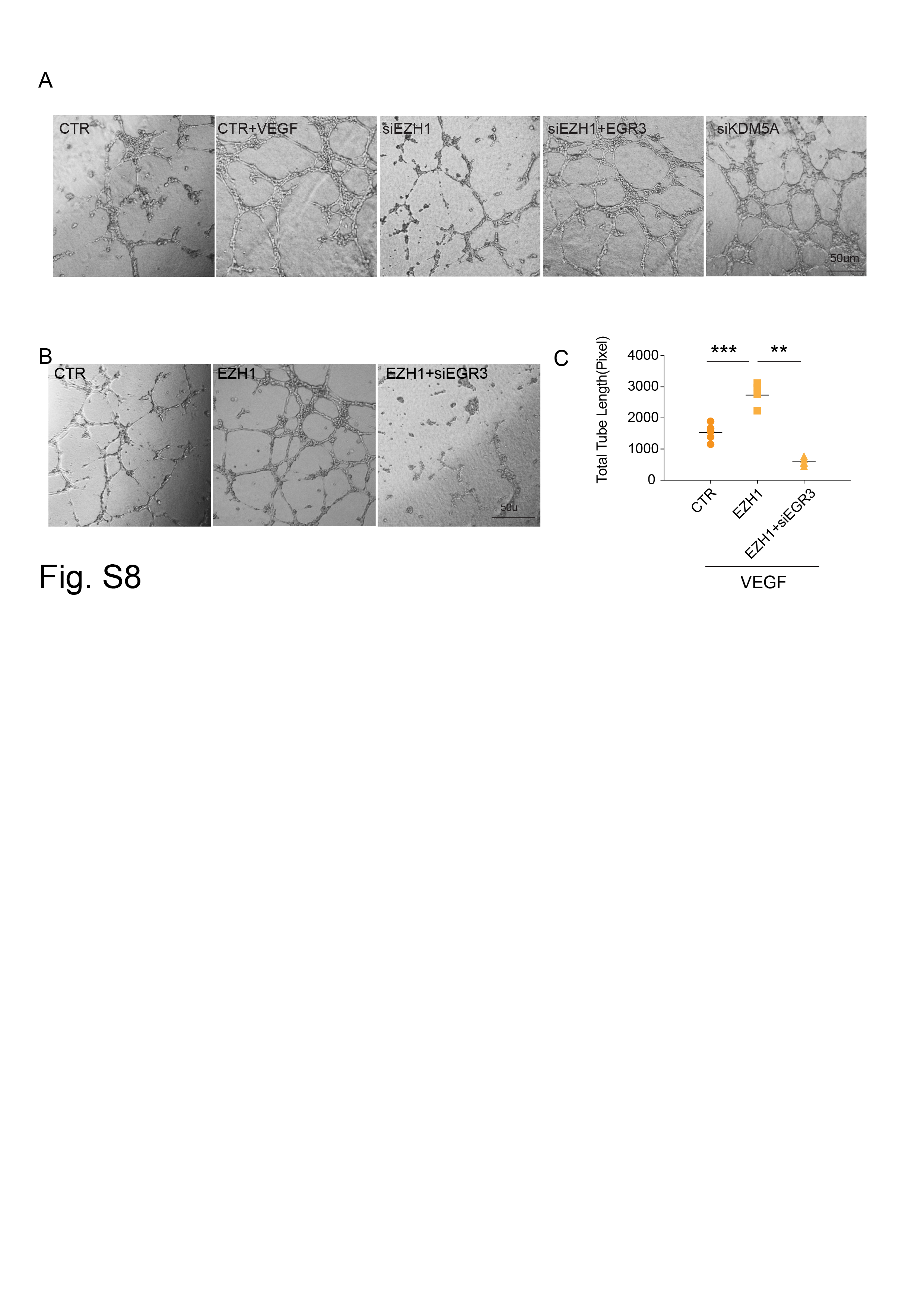

Supplement: Supplementary file 8 — Supplemental figure 8 [file 41419_2020_2228_MOESM8_ESM.png]
