# Supplementary material for: Two faces of bivalent domain regulate VEGFA responsiveness and angiogenesis
Source: Cell Death Dis. 2020 Jan 30;11(1):75. doi: 10.1038/s41419-020-2228-3 (PMC6992747; doi:10.1038/s41419-020-2228-3)
Supplement: Supplementary file 13 — Supplemental Figure Legends [file 41419_2020_2228_MOESM13_ESM.docx]

**Supplemental Figure Legends:**

**Fig. S1 Bivalent domain in the endothelial cells.**

1. Venn diagram showing the overlap between BD called from overlapping H3K4me3 and H3K27me3 peaks and from histone mark cluster C3 and C6; 81.79% of them were overlapped, Fisher’s exact test, *P*-value < 2.2×10^-16^.
2. Gene Expression profiles of BDs (not include bdDEGs), bdDEGs and genes with TSS marked by H3K4me3 but no H3K27me3 (exclude all BDs). Wilcoxon rank-sum test, *P*-value =

2.2×10^-16^, respectively.

1. The tag-density heatmap of variant epigenetic modifications near TSS of bdDEG.

**Fig. S2 Epigenetic traits of bdDEG.**

Average plots of epigenetic marks at early (upregulated expression at 1 hour, left panel) and late (upregulated expression at 4&12 hour, right panel) upregulated bdDEG.

**Fig. S3 VEGF increased EZH1 occupancy to upregulated bdDEG.**

1. VEGF did not change the *EZH1* mRNA expression as shown by RNA-seq.
2. VEGF increased the expression of *EGR3* and *EZH1* but not *KDM5A* and *SUZ12*.

C-E. ChIP-qPCR demonstrated VEGF increased chromatin occupancy of *EZH1* (C) and *SUZ12* (D), and reduced *EZH12* (E) occupancy at the promoters of bdDEG. mean± SD, n=4, unpaired two-tailed Student’s t- test, *: *P*-value < 0.05, **: *P*-value < 0.01. Statistics in summary panel: Mann-whitney U test.

**Fig. S4 Assessment of siRNA knockdown efficiency**

1. Validation of knockdown of *EZH2*, *EZH1*, *KDM5A*, *KDM5B* and *SUZ12* in HUVEC. siRNAs were transfected into HUVEC and gene expression was measured by RT-qPCR after 2 days.
2. Validation of siRNA knockdown of *EZH2, EZH1, KDM5A, KDM5B and SUZ12* in HUVEC. Protein expression was measured by Western blot.
3. *EZH1* lentivirus increased *EZH1* expression in HUVECs.

**Fig. S5 EZH1 enhanced the induction of VEGF regulatory genes in HPVECs.**

1. ChIP-PCR showing the BD also presented at the TSS of bdDEG in HPVECs.
2. RT-qPCR validating the induction of *ADMATS1*, *DLL4*, *EGR3*, *IGFBP3*, *KLF4* and *PROX1* genes upon VEGF treatment with or without knocking down *EZH1*.

**Fig. S6 EZH1 influenced chromatin deposition of H3K27me1 and H3K27me3.**

A-B. EZH1 overexpression increased H3K27me1 (A) but decreased H3K27me3 (B) chromatin deposition at six tested bdDEGs. The right panels are the summarized chromatin occupancy of H3K27me1 and H3K27me3 at all six tested loci. mean± SD, n=4, unpaired two-tailed Student’s t- test, *: *P*-value < 0.05. Mann Whitney test for Summary plots.

**Fig. S7 EZH1 and KDM5A regulated endothelial migration and proliferation by modulating EGR3 expression.**

1. Western blot showing *EGR3* lentivirus increased *EGR3* expression in HUVECs.
2. Western blot validating *EGR3* siRNA inhibited EGR3 protein expression.

C-D. Transwell assay showing *EZH1* and *EGR3* inhibited HUVEC cell migration, while KDM5A did the opposite. C. Migrated HUVECs on the Transwell membrane revealed by crystal violet staining. D. Migrated cells per field. Bar plots: mean± SD, **: *P*-value < 0.01; ***: *P*-value < 0.001. n=6, unpaired two-tailed Student’s *t*-test.

E-F . Transwell assay showing *EZH1* lentivirus enhanced VEGF-induced HPVEC migration, which could be attenuated by knocking down *EGR3*. E. Representative images of migrated HPVECs through the Transwell membrane. F. Cells per fields of migrated cells. Plots: mean± SD, n=6, unpaired two-tailed Student’s *t*-test, ***: *P*-value < 0.001.

G-H. Transwell assay of *EZH1* and *EGR3* knockdown experiments. Knocking down *EZH1* abolished VEGF-induced HPVEC migration, which can be attenuated by the ectopic overexpression of *EGR3*. In contrast, knocking down *KDM5A* enhanced VEGF induced HPVEC migration. G: representative images of migrated HPVECs. H: Cells per field of infiltrated HPVEC. Plots: mean± SD, n=7, unpaired two-tailed Student’s *t*-test, ***: *P*-value < 0.001.

I. upregulation of *EZH1* promoted cell proliferation, *EGR3* siRNA abolished this promotion (I). Bar plots: mean± SD, n=5, unpaired two-tailed Student’s *t*-test, ns: no significance, *: *P*-value < 0.05; **: *P*-value < 0.01.

**Fig. S8 EZH1 and KDM5A regulated endothelial tube formation by modulating EGR3 expression.**

**A.** Representative images of formed endothelial tubes according to Fig. 6D.

**B-C.** *EZH1* promoted endothelial tube formation through the upregulation of *EGR3*. B: Representative images of formed endothelial tubes. C: Calculation of tube length in each tested group. Bar plots: mean± SD, n=4, unpaired two-tailed Student’s *t*-test, **: *P*-value < 0.01, ***: *P*-value < 0.001.
